# Precision Ultrasonic Wave Measurements With Simple Equipment

**DOI:** 10.6028/jres.106.040

**Published:** 2001-10-01

**Authors:** Steven E. Fick, C. Harvey Palmer

**Affiliations:** National Institute of Standards and Technology, Gaithersburg, MD 20899-8221; Johns Hopkins University, Baltimore, MD

**Keywords:** displacement, inexpensive, interferometer, precision, ultrasonic wave

## Abstract

We describe the design and construction of a relatively simple, inexpensive laser interferometer system for accurate measurements of ultrasonic surface displacement waveforms in reasonably friendly environments. We show how analysis of a single waveform can provide both the calibration constant required for absolute measurements and an estimate of the uncertainty of these measurements. We demonstrate the performance of this interferometer by measuring ultrasonic waveforms generated by a novel conical-element ultrasonic transducer.

## 1. Introduction

Ultrasonic methods are now widely used for many purposes: academic, industrial, and medical. For many applications, simple detection suffices to determine the time intervals between pulses. For other uses, such as the determination of material constants, accurate surface displacement waveform measurements may be needed. A variety of systems [1–3] have been shown to yield highly accurate waveform information and to have high sensitivity, even under adverse ambient conditions. Because they typically involve elaborate apparatus: confocal Fabry-Perot interferometers [4,5], photorefractive materials [6], high power laser generators and high power laser detectors, these systems can be very expensive, and can themselves add hazards to the working environment.

Our system, designed for use in reasonably benign environments found in many laboratories, uses relatively inexpensive equipment to yield the desired surface displacement waveform information. It is effectively a point source/point receiver arrangement [7,8]. The ultrasonic wave source is a small conical piezoelectric transducer (designed as an acoustic emission sensor), and the displacement detector is a low power (1 mW) laser interferometer of special design. These, together with associated electronics, a digital oscilloscope, and a computer, compose the system. With it we have been able to obtain high quality, quantitative waveforms.

## 2. Piezoelectric Source Transducer

Our unusual piezoelectric transducer source was developed at the National Bureau of Standards (NBS) [now National Institute of Standards and Technology (NIST)] for use as an acoustic emission sensor [9]. Typical commercial transducers have sensitive areas 10 mm to 25 mm or more in diameter, a scale useful for detecting or generating plane acoustic waves. The NBS design, on the other hand, is optimized for detection of the highly curved wave fronts characteristic of small buried acoustic emission sources. For this purpose, its sensing area is very small—only 0.7 mm in diameter. It can be used effectively as a point receiver because the diameter of the sensing area is small compared to most of the wavelengths to be measured.

**Fig. 1:**
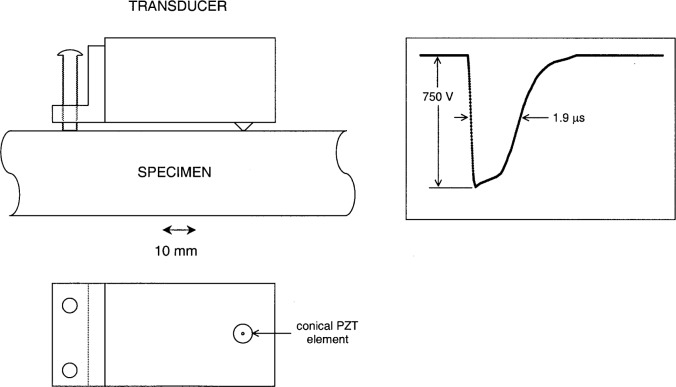
Piezoelectric source transducer and excitation voltage waveform.

The design, illustrated in Fig. 1, incorporates a truncated, conical, lead-zirconate-titanate (PZT) piezoelectric element mounted directly on a large brass block using hard solder. The tip of the element is equipped with a nickel-plated electrode. The specimen itself, if metallic, is used as one of the electrodes of the transducer. With nonmetallic specimens, a thin strip of aluminum foil is interposed between the specimen and the transducer element to provide electrical contact. In either case, the effect of the grounded electrode on the incident elastic wave is much less than that of the wear plate which covers and protects the grounded electrode in transducers of conventional design. The brass block, which substitutes for the usual backing material, is provided with two nylon feet, which, together with the piezoelectric element, provide three-point kinematic support. The weight of the block ensures good contact with the specimen.

The overall design clearly makes the transducer rather delicate and hence not very useful for most commercial applications. However, for acoustic emission sensing in the laboratory and for ultrasonic wave generation, the device has been found to be quite useful.

For the experiments reported here, the transducer was used as a source excited by the 750 V exponential pulse waveform shown in Fig. 1. The excitation pulse was generated by a vacuum tube amplifier driven by a simple exponential pulse generator circuit.

## 3. Interferometer

The classic Michelson interferometer design, in which the sample and reference paths are at right angles and well separated, is especially sensitive to small deflections of the base plate. Furthermore, the presence of air, which under standard conditions has a refractive index of about 1.00029 [10], introduces roughly an extra 45 (slightly shorter) wavelengths in a 100 mm path. Even minute temperature or pressure fluctuations cause the interference fringes to shift significantly. To measure ultrasonic wave details as small as one five thousandth of an optical wavelength, a better design is needed; we used a new interferometer design which was much more satisfactory.

**Fig. 2:**
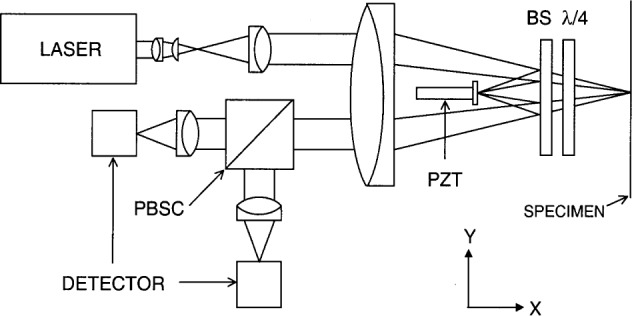
Basic optical system system of the interferometer.

The basic design of our instrument has been described in some detail previously [11]. The essential optical layout is shown in Fig. 2. It features a more-or-less in-line arrangement of both reference and sample beams. The expanded input laser beam is focused by the large lens through the beam splitter plate, BS, onto the specimen. The reference beam is reflected by the beam splitter to focus on the small mirror left of the beam splitter. The focused spot size on the specimen is about 0.02 mm diameter, much smaller than the shortest ultrasonic wavelengths to be measured. If necessary, the spot could be made much smaller by using a lens with shorter focal length. Thus the instrument acts as a point receiver, and neither flatness of the specimen surface nor a high quality optical polish are essential for good results. Non-reflecting specimens were also studied by cementing a tiny mirror on the surface, as explained below. For simplicity, Fig. 2 does not show a small device that redirects the horizontal interferometer beams 90° up or down for probing horizontal surfaces.

The interferometer components are mounted on a long, rigid aluminum U-channel. The fringes are stable, to first order, against any bending of the aluminum base in either the Y or Z directions, or twisting along the X direction, because both sample and reference beams are similarly affected. In addition, with the sample and reference beam parallel over most of their lengths, most small atmospheric changes tend to affect both optical paths about equally.

The interferometer, specimen mount, associated measuring and positioning equipment, pulser, and other components were installed on a heavy, magnetic tabletop originally used for holographic demonstrations. The tabletop in turn was supported by four air-filled inner tubes, which damped out vibrations as low as about 5 Hz. The resulting anti-vibration table in turn was set on a heavy lab bench top supported by four water-filled inner tubes to further damp building vibrations. This homemade arrangement was inexpensive and very effective in suppressing building vibrations.

An advantage of our design over the Michelson design is that virtually no light from either the specimen or the reference mirror is returned to the laser. This isolation removes a potential source of instability in the laser resulting from variable feedback of different amplitude and phase. A more expensive option is to the use a Faraday rotator to isolate the laser.

The use of two photodetectors yields an improvement best explained by first considering how the photodetector output signals for single and dual detector interferometers are similar. For both designs, the output voltage of an individual photodetector can be considered to be the sum of two components: (1) a voltage which is directly proportional to incident optical power (laser power reduced by static losses) but independent of the path difference between reference and sample beams, and (2) a voltage which is determined by both the laser power and the path difference.

Both the path-independent and the path-dependent signal components are affected by variations in laser power. Both signal components can therefore compromise the performance of an interferometer of either design if the frequency range of laser power fluctuations overlaps the frequency range of the signals of interest. With a dual detector system, as in our design, the performance compromise due to the path-independent signal component is eliminated by appropriate manipulation of the amplitudes and phases of both components of the signals from both photodetectors.

The plane of polarization of the incident beam is set at 45° with respect to the vertical. To the right of the beam splitter as shown in Fig. 2, the sample beam passes twice through the suitably oriented quarter wave plate and becomes plane polarized at 90° to the unmodified reference beam. No interference fringes are observable when the two beams are recombined. These beams are now directed to the polarization beam splitter cube, PBSC, which selects the horizontal component of each beam (polarized at 45° and −45° respectively with respect to the vertical and thus out of phase) and directs them to one photodetector. The vertical components (in phase) are directed to the other photodetector. This yields two interference patterns which are 180° out of phase with respect to each other, so that a given change in optical path causes an increase in the intensity of one interference pattern, and a decrease in the intensity of the other interference pattern.

If the reference path is adjusted so that the phase difference between the reference and sample beams is an integer multiple of π/2, the levels of optical power falling on the two photodetectors will be equal. If the gains of the amplifiers following the two photodetectors are adjusted to make their effective sensitivities equal, the output voltages of the two photodetectors will have identical path-independent components, and the path-dependent components will be equal in magnitude but opposite in sign. Subject to these two conditions, if the output voltages of the two photodetectors are subtracted, the path-independent components cancel, while the two path-dependent components add to yield the same signal as would be obtained with a single channel interferometer. This approach reduces the sensitivity of the interferometer output voltage to fluctuations in laser power [12].

**Fig. 3:**
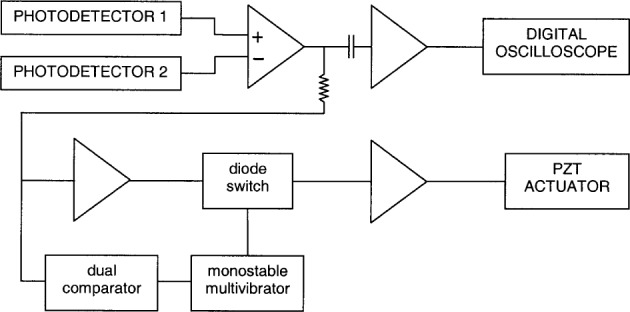
Schematic diagram of control system (DC bias circuitry not shown).

A control system is used to maintain the operating point where the fringe intensities from the two outputs are equal in magnitude (and opposite in phase). For this purpose, the interferometer is provided with a small piezoelectric tube (PZT), 3.2 mm (1/8 in) in diameter and 12.7 mm (1/2 in) long to which the small reference mirror is cemented. This tube is electrically driven to control the path difference. As is shown in Fig. 3, the photodetector output signals are subtracted in a differential amplifier. The high frequency AC ultrasonic component is extracted, amplified, and recorded using a digital oscilloscope with 50 MHz sampling rate. The DC component is amplified and a DC reference voltage subtracted. For clarity, the DC reference source is omitted from the figure. This signal is applied to a high voltage amplifier stage whose output is applied to the PZT actuator supporting the reference mirror. Although most of the circuitry is powered by a well regulated ± 15 V supply, the output stage is powered by a DC supply with output voltage set to either 100 V or 200 V, depending upon whether the interferometer is being used for making measurements or for calibration. The 200 V maximum voltage was chosen to be low enough to preclude damage to the PZT actuator even in the event of a worst-case failure of the output stage.

Over time, the fringe drift can accumulate and cause the DC voltage applied to the PZT actuator to approach the minimum or maximum output of the associated high voltage amplifier. If uncorrected, this phenomenon would latch the control circuit and render it useless. Incipient latch-up is detected by the dual comparator shown at the lower left of Fig. 3. Composed of two symmetrically thresholded hysteresis comparators whose outputs are combined by an OR gate, the dual comparator drives a conventional monostable multivibrator which produces a 1 ms pulse when incipient latch-up occurs. Controlled by this pulse, the diode switch forces the PZT actuator voltage to mid-range for a time long enough to allow the reference mirror to return to the center of its range. This sequence allows the reference mirror to slew to the position required when the feedback loop is re-established at the end of the 1 ms pulse. This control system design was found to be very satisfactory.

## 4. Calibration

An interferometer is said to be calibrated when quantitative knowledge of its characteristics is sufficient to allow values of absolute displacement to be recovered from its raw data. Consisting only of the path-dependent component discussed earlier, the output voltage *V* of our interferometer is given by

$$V=\eta P_{0}\sin\left(4\text{π}\delta /\lambda \right)$$ (1)

where *η* combines the effects of optical losses, photodetector efficiencies, and electronic gains, *P*_0_ represents the laser output power, *δ* is the measured surface displacement in nm, and *λ* is the wavelength of the laser light. The amplitude of the suppressed path-independent component is just *ηP*_0_.

The output voltage *V_m_* of a conventional Michelson interferometer consists of the sum of path-independent and path-dependent components, and is given by

$$V_{m}=\eta P_{0}\left[1+\sin\left(4\text{π}\delta /\lambda \right)\right]$$ (2)

Comparing Eqs. (1) and (2) shows that with our interferometer design, suppression of the path-independent component reduces the effects of in-band laser power fluctuations by the factor sin(4π*δ*/*λ*)/[1 + sin(4 π*δ*/*λ*)].

From Eq. (1) it is clear that an arbitrarily large value of *δ* will result in a bounded value of *V*, and that values of *δ* less than *λ*/8 can be determined unambiguously from values of *V* as accurately as *η*, *P*_0_, and *λ* are known. With the value of *λ* well known, the interferometer can be calibrated using any means which determine *η*, *P*_0_, or their product. Although ways could be devised to determine these parameters separately, it is convenient instead to extract their product from the results of a single measurement.

This can be done by applying a sinusoidal displacement to the reference mirror so that *δ* = *δ*_0_ cos(2π*f*_a_*t*) and the interferometer output voltage is given by

$$V=V_{0}\sin\left[K\delta_{0}\cos\left(2\text{π}f_{a}t\right)\right]$$ (3)

where *δ*_0_ is the maximum displacement of the reference mirror, *V*_0_ = *ηP*_0_ and *K* = 4π/*λ*. This equation represents a rather complicated waveform describable as a series of Bessel functions. It is obvious, however, that one condition for the maximum of the sine function in Eq. (3) to be reached is that *Kδ*_0_ equal an integer multiple of π/2. For small values of *δ*_0_, the appropriate integer multiplier is unity, and the maximum occurs when *δ*_0_ = *λ*/8 or 79.1 nm for the red He-Ne laser light used in our work. For values of *δ*_0_ greater than *λ*/8, the waveform develops regions containing local extremes from which *V*_0_ can be determined easily and unambiguously. This means that the interferometer output voltage can be calibrated in terms of absolute displacement *without* independent knowledge of the displacement used for calibration. This is achieved in practice by driving the PZT actuator supporting the reference mirror with a sinusoidal voltage whose frequency is adjusted to roughly match the fundamental mechanical resonance of the actuator-mirror assembly in order to maximize the displacement of the reference mirror. The dependence of the parameter *η* on specimen surface conditions is taken into account by performing a separate calibration experiment with each specimen, or with each type of specimen if specimens of the same type are independently known to have sufficiently similar surface characteristics. After the resulting calibration waveforms have been analyzed to extract values of *V*_0_, Eq. (1) can be used to find a value of *δ* for any value of *V*.

Sufficiently small values of *δ* invite the use of the sin(*x*) = *x* approximation in Eq. (1). Application of this approximation to Eqs. (1) and (3) shows that

$$\delta =\left(\lambda /4\text{π}V_{0}\right)V$$ (4)

for values of *δ* below an appropriate limit. Since it is best decided in comparison with the other components of measurement uncertainty, this limit is considered in the next section.

## 5. Measurement Uncertainties

All quantitative experimental results are subject to measurement uncertainties due to the performance limits of the equipment used to implement the measurement technique, and due to the effects of phenomena which confound its implementation. Here we consider the magnitudes of the uncertainties of the interferometer as we used it to measure displacement at the specimen surfaces, details of the various confounding phenomena having been considered elsewhere [1–3].

From Eqs. (1) and (3) it is clear that calibration waveforms contain information which describes the performance of the interferometer over its full output voltage range. Since the same information underlies the parameter *V*_0_ which is applied to all other experimental results, the uncertainties applicable to *V*_0_ are essential components of the combined uncertainty applicable to the other experimental results.

We note for the record that, as is apparent from Eq. (4), all experimental results are equally sensitive to changes in *λ* and *V*_0_. For the red He-Ne laser light used in our work, the uncertainties applicable to *λ* are negligible compared to the uncertainties applicable to *V*_0_.

We explore the uncertainties applicable to *V*_0_ by determining the degree to which a typical calibration waveform can be represented by *V* of Eq. (3). As an indicator of the goodness of fit between the experimental calibration waveform and the theoretical waveform from Eq. (3), we choose an easily computed statistic—the sum of the absolute differences between experimental and theoretical voltages for all instants of time represented in the experimental waveform.

The task of calculating the theoretical waveform would be trivial if numerical values for the Eq. (3) parameters *V*_0_, *K*, *δ*_0_, and *f*_a_ were independently known to sufficient accuracy. In practice, only *K* is known *a priori* with high accuracy. The parameter *V*_0_ is to be determined from calibration data, *δ*_0_ can only be approximated by inspection of the calibration waveform, and *f*_a_ is subject to inaccuracies from waveform distortion and signal generator frequency readout.

It is therefore necessary to calculate the theoretical waveform by some other means. We chose to use a conventional spreadsheet program to calculate the theoretical voltage

$$V_{\text{th}}=V_{\text{DC}}+V_{0}\sin\left\{K\delta_{0}\cos\left[2\text{π}f_{a}\left(t+t_{0}\right)\right]\right\}$$ (5)

where *V*_DC_ is the constant required to account for the inevitable DC offset voltage of the interferometer electronics and *t*_0_ is the constant required to account for the arbitrary starting time of each experiment. The spreadsheet was set up with a row for each instant of time represented in the experimental waveform, and columns for time, experimental voltage, the theoretical voltage *V*_th_ computed using a formula, and the absolute value of the difference of the two voltages. Other formulas were used to compute the sum of absolute differences for all instants of time, and the sum of the absolute values of all voltages composing the experimental waveform.

**Fig. 4:**
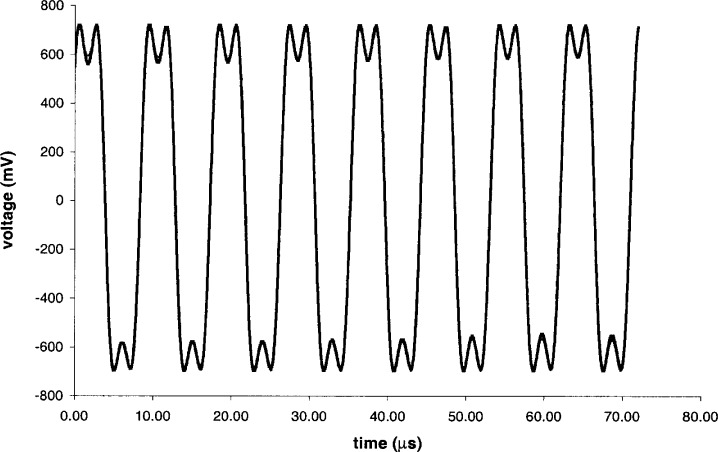
Typical interferometer calibration curve.

We analyzed the waveform shown in Fig. 4, which represents 3609 values sampled at 20 ns intervals. To simplify the analysis, the original 4096-point record was truncated to 8 complete cycles. Analysis of the zero-crossing times reveals that their standard deviation is 216 ns, which is sufficiently large to require that each waveform cycle be analyzed separately.

For each waveform cycle, we iteratively adjusted values for *V*_DC_, *V*_0_, *Kδ*_0_, *f*_a_, and *t*_0_ to minimize the sum of the absolute differences between experimental and theoretical voltages for all instants of time represented in the experimental waveform. The initial value for *V*_0_ was taken from half the difference of the largest and smallest waveform voltages, and the initial value of *f*_a_ was based on the previously calculated zero-crossing times. The five parameters were adjusted sequentially, with each one varied to find a local minimum of the sum of absolute differences. The sequence was repeated with smaller increments for each parameter, until the adjustments changed the value of the sum of absolute differences by less than 0.01 %. As a measure of curve-fitting error for the *i* th cycle, we define a parameter *E_i_* to be the result of dividing the sum of the absolute differences by the sum of the absolute values of the measured voltages themselves.

Each value of *V*_0_ determined using this curve-fitting procedure is subject to an uncertainty due to the finite resolution of the data set representing each cycle of the experimental waveform. In the absence of further information concerning the linearity of our digital oscilloscope, we assume that the true amplitude of a cycle represented by *n* distinct voltage levels will differ from the measured amplitude by no more than one half of the increment between successive voltage levels, and that the consequent fractional uncertainty of the amplitude is therefore 1/2*n*. We define the Type A quantization uncertainty parameter *Q_i_*, expressed in percent, to be equal to 50/*n*, where *n* is the number of distinct voltage levels in the *i* th cycle of the calibration waveform.

In Table 1, which shows the curve fitting results, the subscript *i* distinguishes individual cycle results *V*_0_*_i_* and *δ*_0_*_i_* from the corresponding parameters of Eqs. (3) and (4).

From these results, it is evident that the quantization uncertainty *Q* could be considered to be the dominant influence on the curve-fitting error *E* only for the first few cycles. Careful examination of the waveform, at higher resolution than is practical with the printed figure, reveals the probable cause—electrical noise happened to have much greater effects on the last five cycles than on the first three cycles. This eventuality precludes the modest improvement that could otherwise be achieved by averaging the eight values of *V*_0_*_i_*. Instead, we observe that the results for the first three cycles have the same value for *V*_0_*_i_*, and we base the remaining analysis on these three cycles only. Consequently no arithmetic is needed to determine that *V*_0_ = 699 mV. Because the intended use and small size of this data set do not justify the use of elaborate statistics, simple averaging suffices to determine that *E* = 1.25 %, and *Q* = 0.380 %. By combining *E* and *Q* in quadrature [13], we find that the Type A uncertainty [13] applicable to *V*_0_ is 1.30 %.

With *Q* almost twice the lowest quantization uncertainty possible with our 8 bit oscilloscope, the uncertainty of *V*_0_ could be significantly reduced by modifying the calibration experiment procedure to use a larger fraction of the oscilloscope dynamic range. Further improvements could be made by using waveform averaging during calibration experiments.

Because *V*_0_ will be applied to all other experimental results, it is clear that its uncertainty also applies to all other measurement results. For this reason we define *u*_cal_, the Type A uncertainty due to interferometer calibration, to be the uncertainty of *V*_0_. This definition is conservative (likely to give a result larger than the actual uncertainty) because it attributes to the interferometer any residual effects of the imperfect performance of the signal generator. Results already presented establish that for our interferometer *u*_cal_ = 1.30 %.

It is also clear that each value of *δ* calculated using the sin(*x*) = *x* approximation is subject to a concomitant Type B uncertainty, *u*_sin_, itself calculable in the obvious way. For the given purposes of a particular experiment, calculated values of *u*_sin_ can be used to verify that the use of Eq. (4) is an acceptable alternative to the computationally more burdensome use of Eq. (3) to determine the particular values of *δ*.

Under general circumstances, each measured value of *δ* will be affected by two additional factors—the number of distinct values in the waveform, and the presence of noise, random and otherwise, in the baseline portion of the waveform which ideally would be constant.

For reasons analogous to those already presented with the definition of *Q_i_*, we now define *u*_q_, the Type A quantization uncertainty in percent of a measured value of δ, to be 50 times the reciprocal of the number of increments of displacement represented by *δ*. For example, for a hypothetical waveform whose increment of displacement is 0.01 nm, *u*_q_ would be 0.5 % for *δ* = 1 nm.

We define *u*_n_, the Type A uncertainty in percent due to the noise in the baseline of a particular set of values of *δ* composing a waveform, to be 100 times the root-mean-square average of the differences between each value in the baseline and the mean of all values in the baseline, divided by the mean of all values in the baseline.

The definitions and embedded statistical procedures for all uncertainty components considered in this paper were chosen to describe the performance of our simple, inexpensive interferometer in general purpose experiments. Data from more sophisticated interferometers designed for specific applications are subject to other and more numerous measurement uncertainties whose characterization requires methods and statistical techniques far more elaborate than those described here.

## 6. Nanometer-Scale Waveforms

Here we present experimental waveforms to demonstrate the performance of our interferometer measuring displacement amplitudes on the order of 1 nm. For these measurements, the piezoelectric source transducer was located at the center of the top surface of a polished 6061T6 aluminum alloy disk 178 mm in diameter, 31.4 mm thick, and positioned with its axis of rotation vertical. The interferometer beam was located at the epicenter and various other points on the bottom surface of the specimen.

**Fig. 5:**
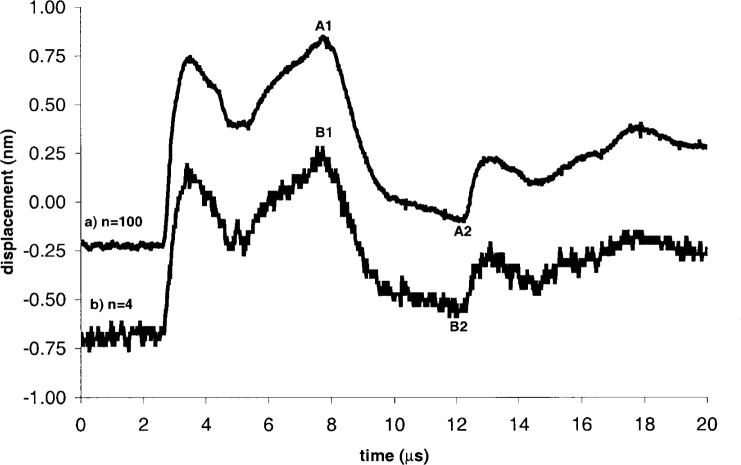
Epicenter waveforms showing effect of averaging.

Two waveforms measured at the epicenter are shown in Fig. 5, displaced vertically for easier viewing. The upper waveform (Fig. 5a) is the result of averaging 100 consecutively captured waveforms, and the lower waveform (Fig. 5b) is the result of averaging 4 consecutively captured waveforms. The benefit of averaging the additional waveforms is visually apparent. The four waveform features marked A1, A2, B1, and B2 represent the largest and smallest excursions from the baselines of their respective waveforms.

**Fig. 6:**
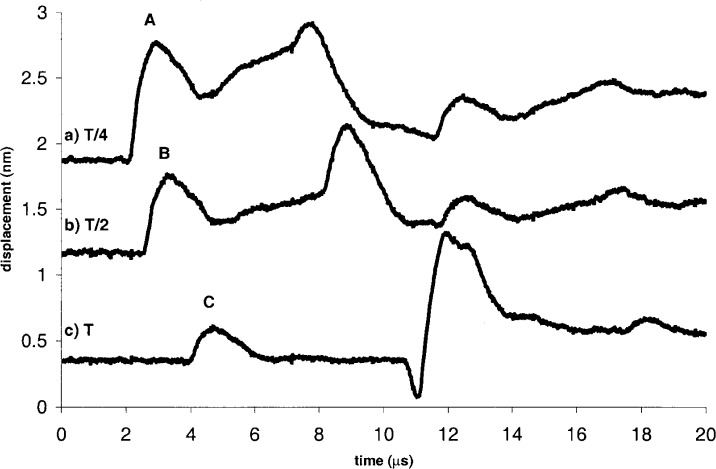
Waveforms for 3 locations with source and receiver on same side of specimen.

Figure 6 shows the results of opposite-side measurements at three distances from epicenter, each a submultiple of *T*, the plate thickness. Each waveform is the result of averaging 100 consecutively captured waveforms. For clarity, the waveforms in the figure have been offset vertically. The three waveform features marked A, B, and C were chosen as representative of the range of displacements in these waveforms.

Table 2 presents the displacements and corresponding uncertainties for the seven marked points in Figs. 5 and 6. Data are grouped by the number of waveforms averaged, with data for 100-waveform averaging preceding data for 4-waveform averaging. Within each group, data are listed in descending order of displacement Δ*δ* measured relative to the baseline. Data for noise and the number of levels were computed according to the definitions already given for *u*_n_ and *u*_q_. Computed by taking the quadrature sum of *u*_sin_, *u*_n_, *u*_q_, and *u*_cal_, the parameter *u_δ_* is the combined relative standard uncertainty [13] (in percent) for an individual value of *δ* in general, and also for Δ*δ* as defined here, because the subtracted baseline consists of an AC component taken into account by *u*_n_ and a DC component added to control vertical position in the figure. Separate columns for the Type A and Type B components of *u_δ_* are not shown because the effect of *u*_sin_, the only Type B uncertainty, is insignificant compared to the effects of *u*_n_, *u*_q_, and *u*_cal_.

Reading down the column for *u_δ_*, it is clear that *u_δ_* increases with decreasing Δ*δ*, and that *u*_n_ is the uncertainty component controlling the trend, as would be expected in the absence of unusual circumstances. By comparing 4-waveform and 100-waveform values of *u*_n_ for similar values of Δ*δ*, it is evident that the reduction in *u*_n_ is less than the factor of five predicted by the Central Limit theorem, as would be expected for a system with numerous noise sources [1–3]. The tabulated data also show that, even with inexpensive equipment, 20 MHz bandwidth dynamic absolute displacement can be measured with combined standard uncertainties in the tens of picometers.

The availability of measured values of path difference allows quantitative evaluation of the benefit of suppression of the path-independent output signal component. Recalling the result presented after the introduction of Eq. (2), it is easy to calculate that, for the 0.128 nm to 1.063 nm range of path differences shown in Table 2, the potential effect of in-band laser power fluctuations is only 0.3 % to 2.1 % as large as the same effect would have been with a conventional Michelson interferometer. For the much larger 108 nm to 110 nm path differences used for calibration, the similar ratios range from 26 % to 27 %. Therefore significant improvement is demonstrated even for the largest displacements likely to be measured.

## 7. Conclusion

In this paper we have shown that it is possible to obtain accurate waveforms and amplitudes together with accurate timing with the use of relatively inexpensive equipment. Obviously, the design and construction of the various parts—interferometer, piezoelectric source transducer, electronics, and mounting assemblies—required a substantial level of effort. However, most of the components could be made by skillful students under proper guidance. Suitable digital oscilloscopes are now inexpensive enough to be kept on hand for general purpose use, and adequate personal computers are now ubiquitous. The necessary data processing could be accomplished using even the most inexpensive readily available spreadsheet software. We have shown how simple calculations can be used to construct a detailed estimate of the uncertainties associated with the use of the interferometer to measure repetitive waveforms typical of those used for wave propagation and simulated acoustic emission studies.

## Figures and Tables

**Table 1:** Calibration waveform curve-fitting results

Cycle (*i*)	*V*_0_*_i_* (mV)	*δ*_0_*_i_* (nm)	*E_i_* (%)	*Q_i_* (%)	Data points
1	699	108.0	1.04	0.403	451
2	699	108.6	1.29	0.376	447
3	699	109.0	1.40	0.360	448
4	701	109.3	1.87	0.379	447
5	700	110.0	2.58	0.379	448
6	703	110.1	3.54	0.357	448
7	702	110.0	4.60	0.385	446
8	704	109.5	4.30	0.376	448

**Table 2:** Displacements and uncertainties for 7 sample points from 5 waveforms

Point	No. of avgs.	Δ*δ* (nm)	Noise (nm)	Levels	*u*_sin_ %	*u*_n_ %	*u*_q_ %	*u*_cal_ %	*u_δ_* %	*u_δ_* (nm)
Fig. 5a A1	100	1.063	0.009	380	0.0074	0.83	0.13	1.30	1.55	0.016
Fig. 6 A	100	0.889	0.008	80	0.0052	0.85	0.63	1.30	1.67	0.015
Fig. 6 B	100	0.572	0.011	87	0.0021	2.00	0.57	1.30	2.46	0.014
Fig. 6 C	100	0.230	0.008	147	0.0003	3.57	0.34	1.30	3.81	0.009
Fig. 5a A2	100	0.125	0.009	41	0.0001	7.07	1.22	1.30	7.29	0.009
Fig. 5b B1	4	0.935	0.031	158	0.0057	3.27	0.32	1.30	3.53	0.033
Fig. 5b B2	4	0.128	0.031	39	0.0001	23.95	1.28	1.30	24.02	0.031
